# Two-color fluorescent *in situ *hybridization in the embryonic zebrafish brain using differential detection systems

**DOI:** 10.1186/1471-213X-11-43

**Published:** 2011-07-04

**Authors:** Gilbert Lauter, Iris Söll, Giselbert Hauptmann

**Affiliations:** 1 Karolinska Institutet, Department of Biosciences and Nutrition, SE-141 83 Huddinge, Sweden

**Keywords:** digoxigenin, dinitrophenol, tyramide signal amplification (TSA), dextran sulfate, hydrogen peroxide, permeabilization, Fast Red, Fast Blue, alkaline phosphatase, horseradish peroxidase, prosomere

## Abstract

**Background:**

Whole-mount *in situ *hybridization (WISH) is extensively used to characterize gene expression patterns in developing and adult brain and other tissues. To obtain an idea whether a novel gene might be involved in specification of a distinct brain subdivision, nucleus or neuronal lineage, it is often useful to correlate its expression with that of a known regional or neuronal marker gene. Two-color fluorescent *in situ *hybridization (FISH) can be used to compare different transcript distributions at cellular resolution. Conventional two-color FISH protocols require two separate rounds of horseradish peroxidase (POD)-based transcript detection, which involves tyramide signal amplification (TSA) and inactivation of the first applied antibody-enzyme conjugate before the second detection round.

**Results:**

We show here that the alkaline phosphatase (AP) substrates Fast Red and Fast Blue can be used for chromogenic as well as fluorescent visualization of transcripts. To achieve high signal intensities we optimized embryo permeabilization properties by hydrogen peroxide treatment and hybridization conditions by application of the viscosity-increasing polymer dextran sulfate. The obtained signal enhancement allowed us to develop a sensitive two-color FISH protocol by combining AP and POD reporter systems. We show that the combination of AP-Fast Blue and POD-TSA-carboxyfluorescein (FAM) detection provides a powerful tool for simultaneous fluorescent visualization of two different transcripts in the zebrafish brain. The application of different detection systems allowed for a one-step antibody detection procedure for visualization of transcripts, which significantly reduced working steps and hands-on time shortening the protocol by one day. Inactivation of the first applied reporter enzyme became unnecessary, so that false-positive detection of co-localization by insufficient inactivation, a problem of conventional two-color FISH, could be eliminated.

**Conclusion:**

Since POD activity is rather quickly quenched by substrate excess, less abundant transcripts can often not be efficiently visualized even when applying TSA. The use of AP-Fast Blue fluorescent detection may provide a helpful alternative for fluorescent transcript visualization, as the AP reaction can proceed for extended times with a high signal-to-noise ratio. Our protocol thus provides a novel alternative for comparison of two different gene expression patterns in the embryonic zebrafish brain at a cellular level. The principles of our method were developed for use in zebrafish but may be easily included in whole-mount FISH protocols of other model organisms.

## Background

*In situ *hybridization is the method of choice to characterize the spatial distribution of gene transcripts during embryonic development as well as in adult tissues. Initial protocols used isotope-labeled nucleotide probes for detection of transcripts on tissue sections [1]. A major methodological advance was the introduction of non-radioactive digoxigenin-labeled probes that permitted for the first time to visualize global gene expression patterns in *Drosophila *embryos [2]. This set the starting point for detection of global transcript distributions in complete tissues, organs and embryos of invertebrate and vertebrate model species. Another milestone was the development of multicolor whole-mount *in situ *hybridization (WISH) procedures for differential color visualization of two or more mRNAs in one and the same embryo [3-7]. In the original method for zebrafish embryos, digoxigenin- and fluorescein-labeled RNA probes were together hybridized and sequentially visualized by two rounds of alkaline phosphatase (AP) detection using Fast Red and BCIP/NBT as differential colorimetric substrates [8,9]. This protocol has been used to compare numerous regulatory gene expression domains in the developing zebrafish brain [10-14].

Fast Red forms a red precipitate, which can be fluorescently visualized using Texas Red or rhodamine filter sets [15]. Fast Red in combination with ELF (enzyme labeled fluorescence) substrate [16] has been used for initial tries of two-color fluorescent *in situ *hybridization (FISH) based on AP detection in zebrafish and mouse [17,18]. However, the low sensitivity and speckled signal of the ELF substrate did not produce satisfactory results, so that a second powerful fluorescent AP substrate for whole-mount FISH was missing. Therefore, current whole-mount FISH protocols instead apply horseradish peroxidase (POD) and fluorescent tyramide substrates for signal amplification [19]. The signal enhancement in combination with the availability of a number of different fluorescent tyramide substrates made multicolor whole-mount FISH possible [20-25]. However, POD is inactivated by substrate excess, so that enzymatic activity is rather quickly quenched. Consequently, the tyramide signal amplification (TSA) reaction can last productively only for less than 30 minutes. This is often not sufficient for detection of lower expressed transcripts. In contrast, AP-based substrate turn over can last over hours because of long-lasting enzymatic activity and high signal-to-noise ratio. Furthermore, because of relatively high background autofluorescence of zebrafish embryos and substrate trapping in the hydrophobic yolk the introduction of the TSA system into multiplex FISH applications for this model organism was problematic. As a consequence, a current protocol tried to compensate for the lowered sensitivity by using additional layers of antibody detection for signal amplification, which made the procedure even longer and more laborious [20].

To make use of the benefits of both, long-lasting enzymatic activity of AP as well as tyramide signal amplification of POD, we aimed to combine the two detection systems for two-color whole-mount FISH. We show here that aside Fast Red also Fast Blue [4,26] produces chromogenic as well as fluorescent signals. To increase signal strength of Fast dyes, we optimized embryo permeabilization properties and hybridization efficiency, respectively, by hydrogen peroxide treatment and addition of dextran sulfate to the hybridization reaction. The resulting increased signal intensities allowed us to combine AP-Fast dye and POD-TSA detection for two-channel fluorescent visualization of different mRNA probes. Because of the different reporter systems applied only one antibody incubation step was necessary and an antibody-enzyme conjugate inactivation step could be omitted resulting in reduced working steps and time spent.

## Results and discussion

### Effect of dextran sulfate in AP-based chromogenic WISH

**Figure 1:**
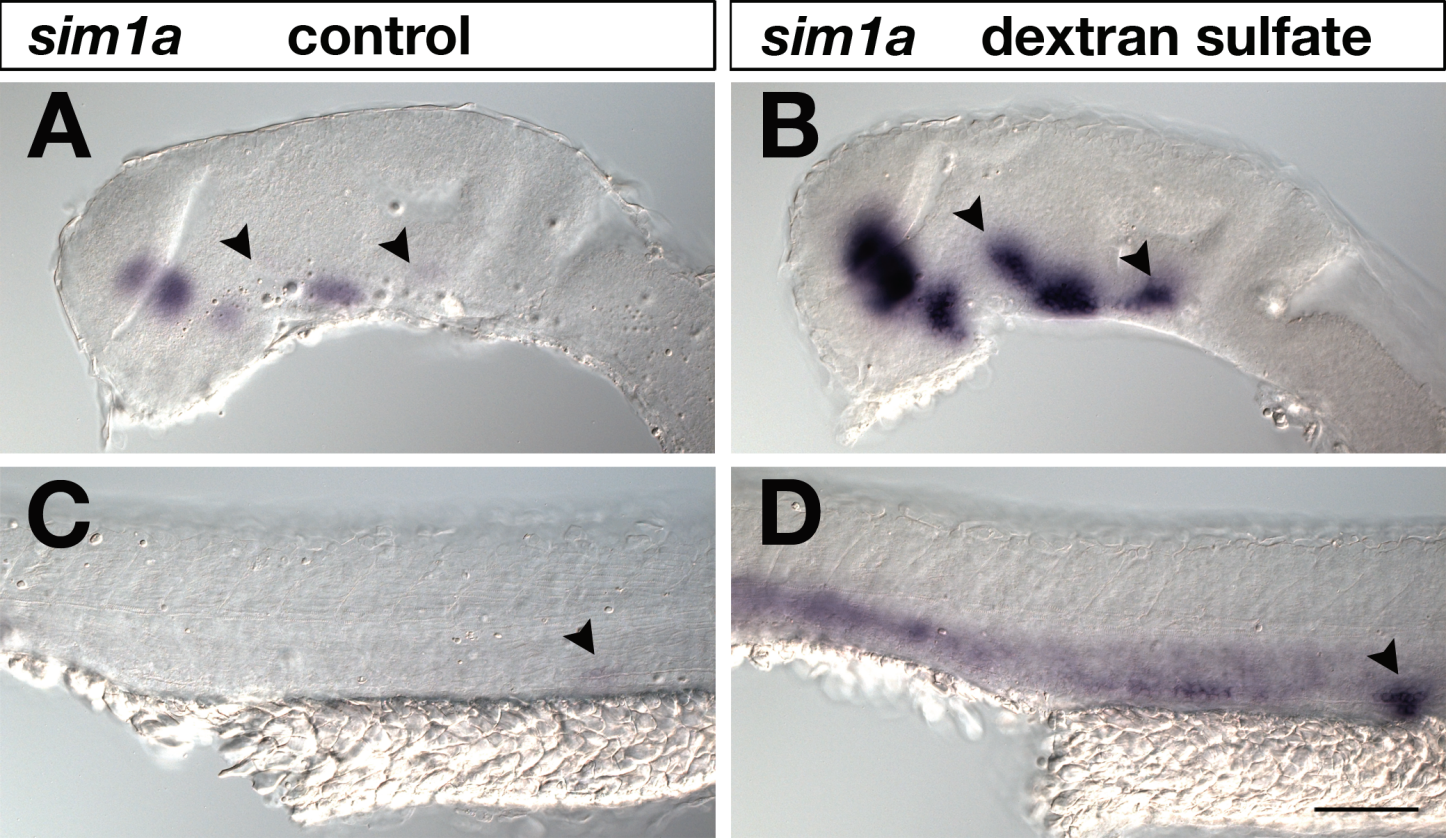
**Effects of dextran sulfate in standard WISH**. 24-hpf embryos were hybridized to a *sim1a *digoxigenin-labeled RNA probe, which was visualized by AP-BCIP/NBT chromogenic detection under identical conditions and staining times. Lateral views of embryonic brains (A,B) and trunks (C,D) are shown with anterior to the left. In (B,D) but not in (A,C) 5% dextran sulfate was included in the hybridization buffer. Addition of dextran sulfate resulted in increased signal sensitivity. Arrowheads in (A,B) indicate *sim1a*-positive neuronal clusters identified in dextran sulfate treated brains (B) that were hardly detected in untreated embryos (A). Arrowheads in (C,D) mark the pronephric primordium strongly visualized in dextrane sulfate treated embryos (D) but not in untreated specimens (C). Embryos were viewed on an Axioplan II microscope and images were recorded with an Axiocam digital camera. Scale bar is 100 μm.

An essential component of successfully performing a WISH experiment is to achieve reasonably high signal sensitivities. Since addition of viscosity-increasing polymers could improve POD-based FISH signals [25,27], we tested in a pilot experiment whether addition of dextran sulfate to the hybridization reaction would improve signal sensitivity in AP-based WISH. 24-hpf zebrafish embryos were hybridized to a *sim1a*-specific digoxigenin probe with or without addition of 5% dextran sulfate to the hybridization mix and transcripts were visualized by AP-based BCIP/NBT staining under identical conditions and staining times (Figure 1). Expression of *sim1a *in the embryonic rostral brain was detected by both samples, but expression sites were much stronger visualized in dextran sulfate treated embryos (Figure 1A,B). Less pronounced expression sites in the basal brain and pronephric primordium could easily be missed in embryos hybridized without dextran sulfate addition (arrowheads in Figure 1A,C) as compared to dextran-treated embryos (Figure 1B,D). The positive effect of dextran sulfate is probably due to a molecular crowding effect that may lead to a local increase of probe concentration [28].

### Optimization of Fast dye signal strength

**Figure 2:**
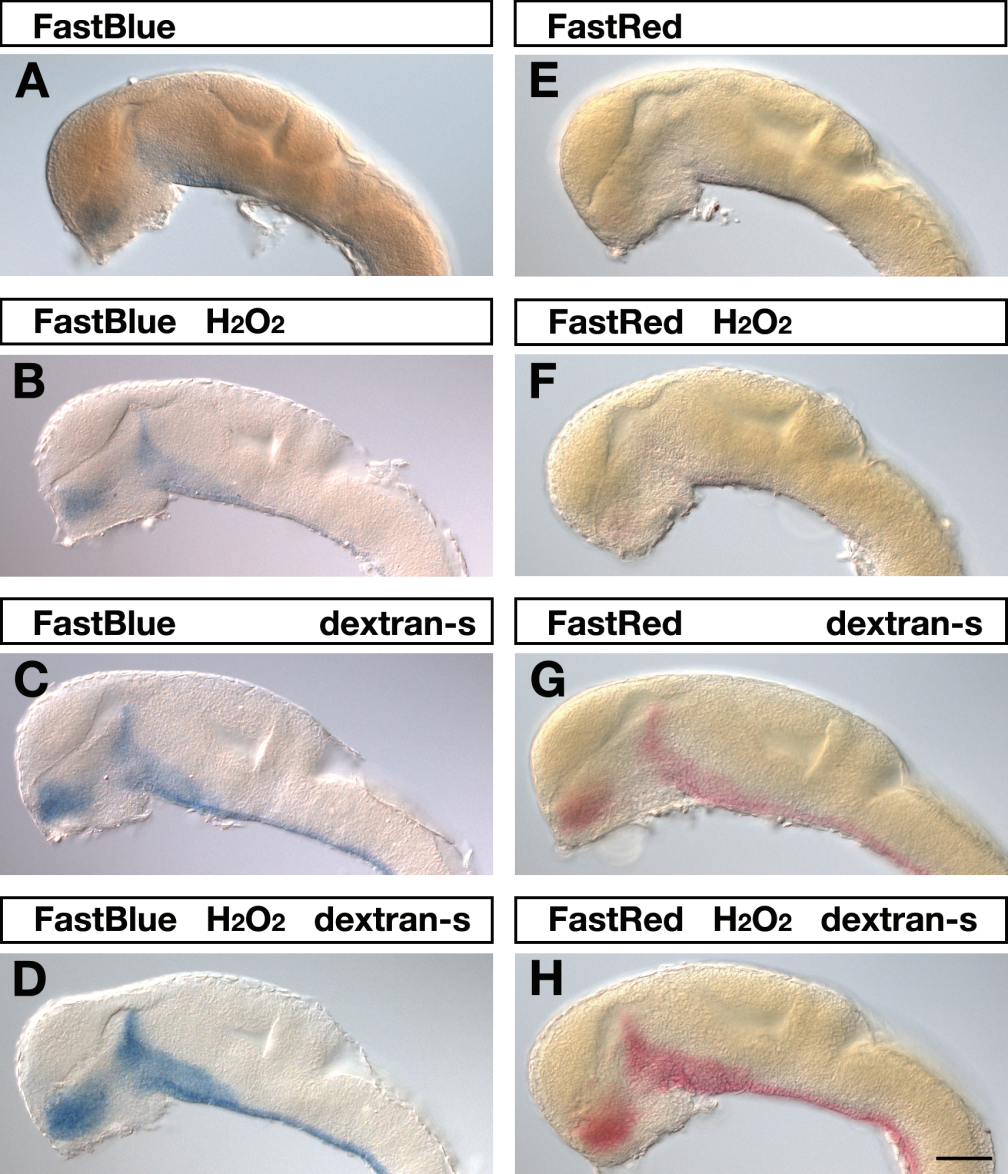
**Effects of hydrogen peroxide and dextran sulfate on Fast Red and Fast Blue detection**. 24-hpf embryos were hybridized to a *shha *dinitrophenyl-labeled RNA probe, which was visualized by AP-Fast Blue (A-D) or AP-Fast Red (E-H) chromogenic staining for 55 min and 80 min, respectively. Lateral views of embryonic brains are shown with anterior to the left. On top of each panel it is indicated whether embryos were permeabilized with 2% hydrogen peroxide (H_2_O_2_) and whether 5% dextran sulfate (dextran-s) was added to the hybridization buffer. Strongest signals were obtained when hydrogen peroxide treated embryos were hybridized in the presence of dextran sulfate (D,H). Embryos were viewed on an Axioplan II microscope and images were recorded with an Axiocam digital camera. Scale bar is 100 μm.

We previously showed that Fast Red and Fast Blue could be applied to chromogenic visualization of transcripts in WISH experiments in *Drosophila *and zebrafish embryos [4,26,29]. For two-color experiments it is essential to achieve high signal intensities and signal-to-noise ratios with both probes. However, Fast dyes are less sensitive than standard BCIP/NBT staining. We therefore tested whether presence of dextran sulfate in the hybridization mix would improve signal intensity when using Fast Red or Fast Blue as substrates. 24-hpf zebrafish embryos were hybridized to a dinitrophenol-labeled antisense RNA probe specific for *shha *and visualized using Fast Red or Fast Blue as substrates (Figure 2). Both, Fast Red and Fast Blue substrate deposition was dramatically increased in dextran sulfate treated (Figure 2C,G) as compared to untreated embryos (Figure 2A,E) under otherwise identical conditions and staining times.

To further improve signal sensitivities we aimed to improve accessibility of embryos for probes and antibody-enzyme conjugates. In previous experiments we made use of hydrogen peroxide to block endogenous peroxidase activity of embryos. We thereby noticed that this treatment lead to improved signal intensities. Because hydrogen peroxide can disrupt cell membranes, we sought to explore the possibility whether hydrogen peroxide would be useful to improve embryo permeabilization properties. Prior to standard proteinase K digestion, we treated the fixed embryos with 2% hydrogen peroxide. Hydrogen peroxide treatment resulted in slightly improved signal detection (Figure 2B,F) and when hydrogen peroxide permeabilized embryos were hybridized in the presence of dextran sulfate strongest signal intensities were obtained (Figure 2D,H).

**Figure 3:**
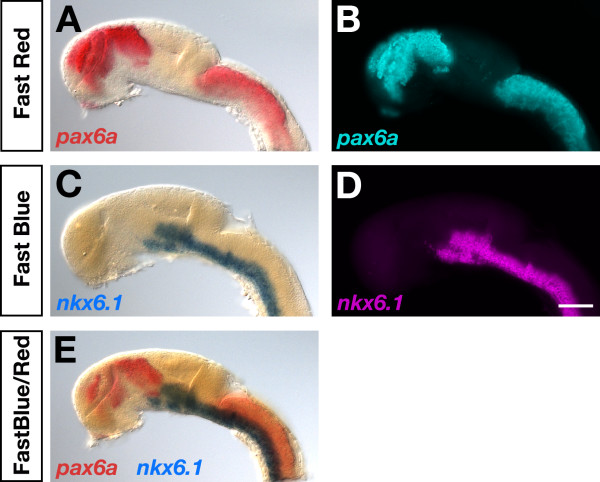
**Fast Red and Fast Blue permit chromogenic and fluorescent transcript visualization**. Lateral views of embryonic brains at 24 hpf hybridized to *nkx6.1 *digoxigenin and/or *pax6a *dinitrophenol antisense RNA probes as indicated on each panel. Transcript distributions were visualized by chromogenic (A,C,E) or fluorescent (B,D) detection of Fast Blue (C,D,E) and Fast Red (A,B,E) precipitates as indicated. Embryos were viewed on an Axioplan II microscope and images were recorded with an Axiocam digital camera. Fluorescent signals were false-colored in ImageJ. Scale bar is 100 μm.

Next, we applied Fast Blue in combination with Fast Red to chromogenic two-color detection of *nkx6.1 *and *pax6a *(Figure 3E). Expression of *nkx6.1 *was visualized by AP-Fast Blue staining. After inactivation of the first applied antibody-AP conjugate, *pax6a *expression was revealed by AP-Fast Red chromogenic reaction. In AP-based two-color experiments the second detection round is less sensitive than the first one, so that the positive effects of hydrogen peroxide for improved embryo permeabilization were even more obvious. Chromogenic detection of *pax6a *transcripts by AP-Fast Red as second round staining revealed that an optimal signal was obtained within 4 hours staining time in hydrogen peroxide permeabilized embryos. In contrast, embryos that were not permeabilized with hydrogen peroxide developed barely detectable chromogenic signals within the same staining time. Comparably strong signals were however obtained after prolonged Fast Red staining of 12 hours (not shown).

### Chromogenic and fluorescent WISH using Fast dyes as AP substrates

Fast Red forms a red precipitate that can be fluorescently visualized using rhodamine filter sets as exemplified here by chromogenic and fluorescent detection of *pax6a *transcript distribution in the forebrain and hindbrain at 24 hpf (Figure 3A,B). To test whether Fast Blue is comparably useful for fluorescent mRNA detection, we hybridized 24-hpf zebrafish embryos with an *nkx6.1*-specific digoxigenin probe followed by AP-Fast Blue detection (Figure 3C,D). Expression of *nkx6.1 *in the basal fore- and midbrain and along the ventral hindbrain and spinal cord could be clearly shown by Fast Blue precipitate deposition (Figure 3C) as well as fluorescent visualization using far-red filter sets (Figure 3D). Fluorescent detection (Figure 3D) revealed corresponding transcript distributions as those obtained with chromogenic substrate deposition (Figure 3C) confirming the specificity of the fluorescent signal.

It is certainly possible to combine Fast Blue and Fast Red for chromogenic two-color WISH [4,26,29] and we revealed clearly abutting expression patterns of *nkx6.1 *and *pax6a *in the experiment presented (Figure 3E). However, two-color fluorescent visualization of transcripts cannot be recommended using both Fast dyes together, since Fast Red and Fast Blue display overlapping red fluorescence emission, leading to significant bleed-through between channels (data not shown). In addition, the two chromogenic precipitates formed during the AP-reaction can obscure each other's fluorescent signal. Therefore, it may be more advantageous to combine AP-Fast dye and POD-TSA fluorescent detection systems.

### Combination of AP and POD systems

We tested the versatility of combining AP and POD substrate reactions for fluorescent detection of different transcripts. A prerequisite of using Fast dyes in two-color FISH is to identify whether there is bleed-through [30] between the chosen detection windows. Since zebrafish embryos have significantly high cyan and UV, but rather low red and green autofluorescence [20], we sought to best combine Fast dye and green TSA-carboxyfluorescein (FAM) for two-channel fluorescence detection.

**Figure 4:**
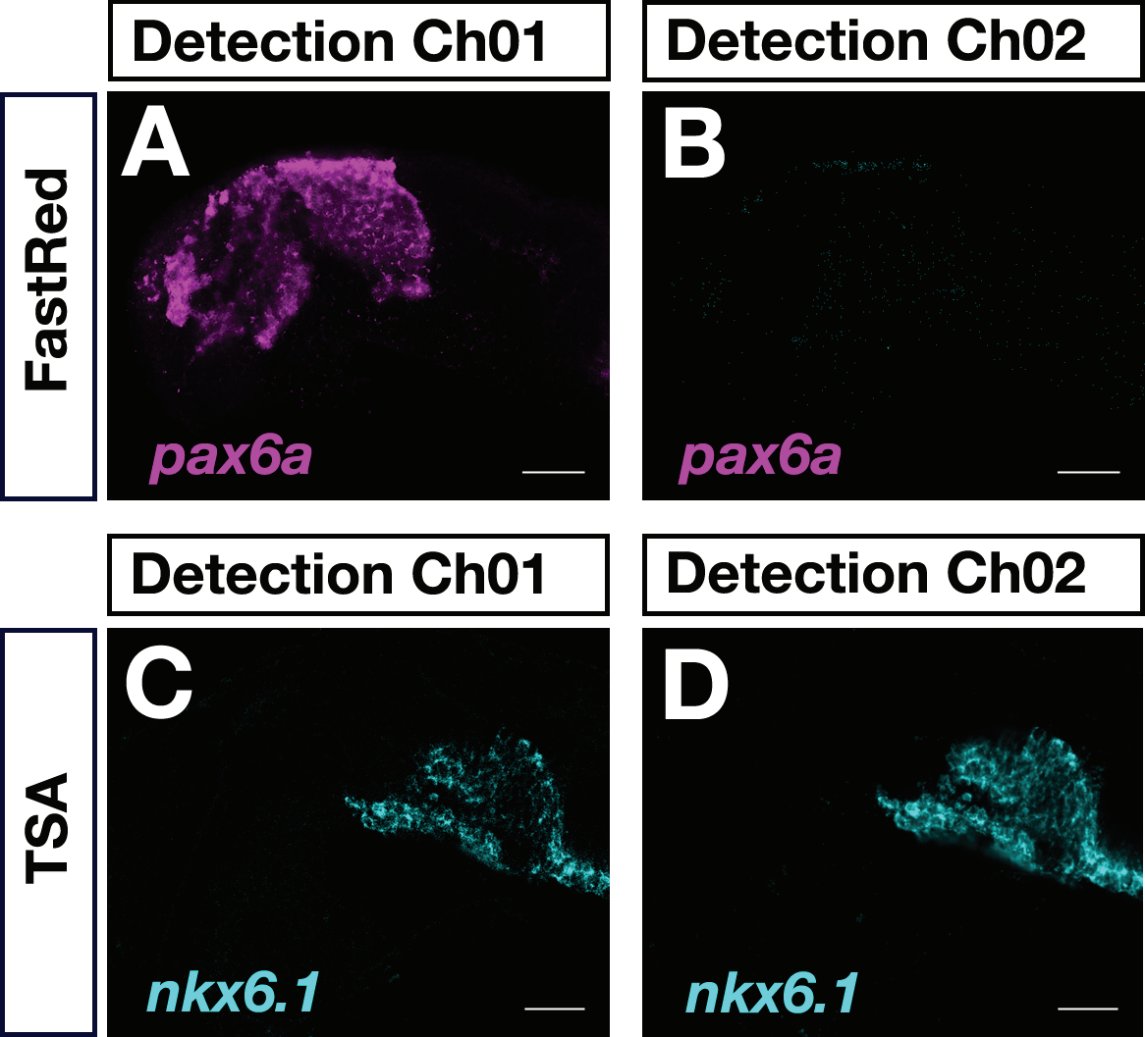
**Control of bleed-through between Fast Red and TSA-FAM detection channels**. 24-hpf embryos were hybridized to a dinitrophenol-labeled *pax6a *probe (A,B) or to a digoxigenin-labeled antisense RNA probe specific for *nkx6.1 *(C,D). Transcripts were detected using Fast Red (A,B) and TSA-FAM (C,D). Fluorescence signals were recorded in the Fast Red (Ch01: detection of wavelengths greater than 560 nm) and TSA-FAM (Ch02: detection of wavelengths from 505 nm to 545 nm) detection channels. A significant bleed-through of TSA-FAM *nkx6.1 *signal was detected in the Fast Red detection channel (C). Lateral views with anterior to the left are shown. Images were recorded on a LSM510 confocal microscope and false-colored in ImageJ. Scale bar is 50 μm.

In single-color FISH experiments, *pax6a *and *nkx6.1 *were visualized by Fast Red and TSA-FAM fluorescence detection, respectively, and each expression pattern was recorded in both detection channels (Channel01: wavelengths greater than 560 nm were collected; Channel02: wavelengths between 505 nm and 545 nm were collected). Forebrain expression of *pax6a *was appropriately visualized in the Fast Red (Ch01; Figure 4A) and not in the TSA-FAM detection channel (Ch02; Figure 4B). In contrast, *nkx6.1 *expression in the fore- and midbrain was visualized in both, the TSA-FAM (Figure 4D) and the Fast Red (Figure 4C) detection channel, although somewhat weaker in the latter one. This indicated significant bleed-through of TSA-FAM fluorescence into the Fast Red channel. These results strongly suggested that caution had to be taken to exclude false-positive co-localization, when choosing these two substrates in a two-color FISH experiment.

**Figure 5:**
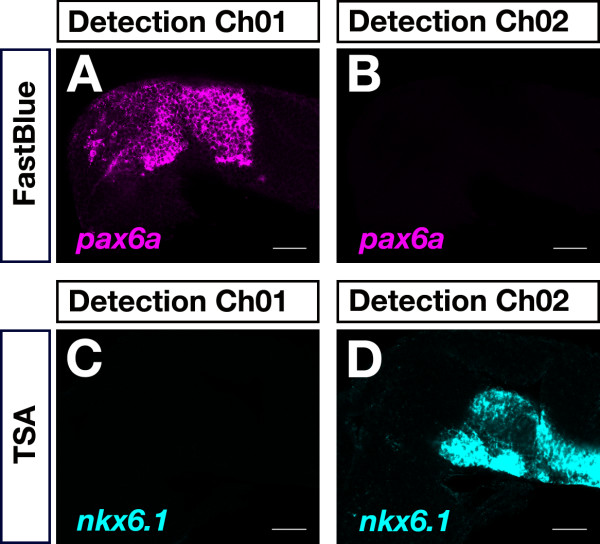
**Control of bleed-through between Fast Blue and TSA-FAM detection channels**. 24-hpf embryos were hybridized to a dinitrophenol-labeled *pax6a *probe (A,B) or to a digoxigenin-labeled antisense RNA probe specific for *nkx6.1 *(C,D). Transcripts were detected using Fast Blue (A,B) and TSA-FAM (C,D). Fluorescence signals were recorded in the Fast Blue (Ch01: detection of wavelengths greater than 650 nm) and TSA-FAM (Ch02: detection of wavelengths from 505 nm to 545 nm) detection channels. No bleed-through was detected between the TSA-FAM and Fast Blue detection channels (B,C). Lateral views with anterior to the left are shown. Images were recorded on a LSM510 confocal microscope and false-colored in ImageJ. Scale bar is 50 μm.

There was no bleed-through observed, however, between the Fast Blue and TSA-FAM detection channels (Channel01: wavelengths greater than 650 nm were collected; Channel02: wavelengths between 505 nm and 545 nm were collected). Detection of *pax6a *by Fast Blue revealed a specific signal in the appropriate (Ch01; Figure 5A) but not in the TSA-FAM detection channel (Ch02; Figure 5B). Despite a very strong *nkx6.1 *TSA-FAM signal was visualized (Ch02; Figure 5D), it could not be detected in the Fast Blue detection channel (Ch01; Figure 5C). These results demonstrated the versatility of combining Fast Blue and TSA-FAM for two-color FISH.

### Application of Fast dyes in two-color FISH

**Figure 6:**
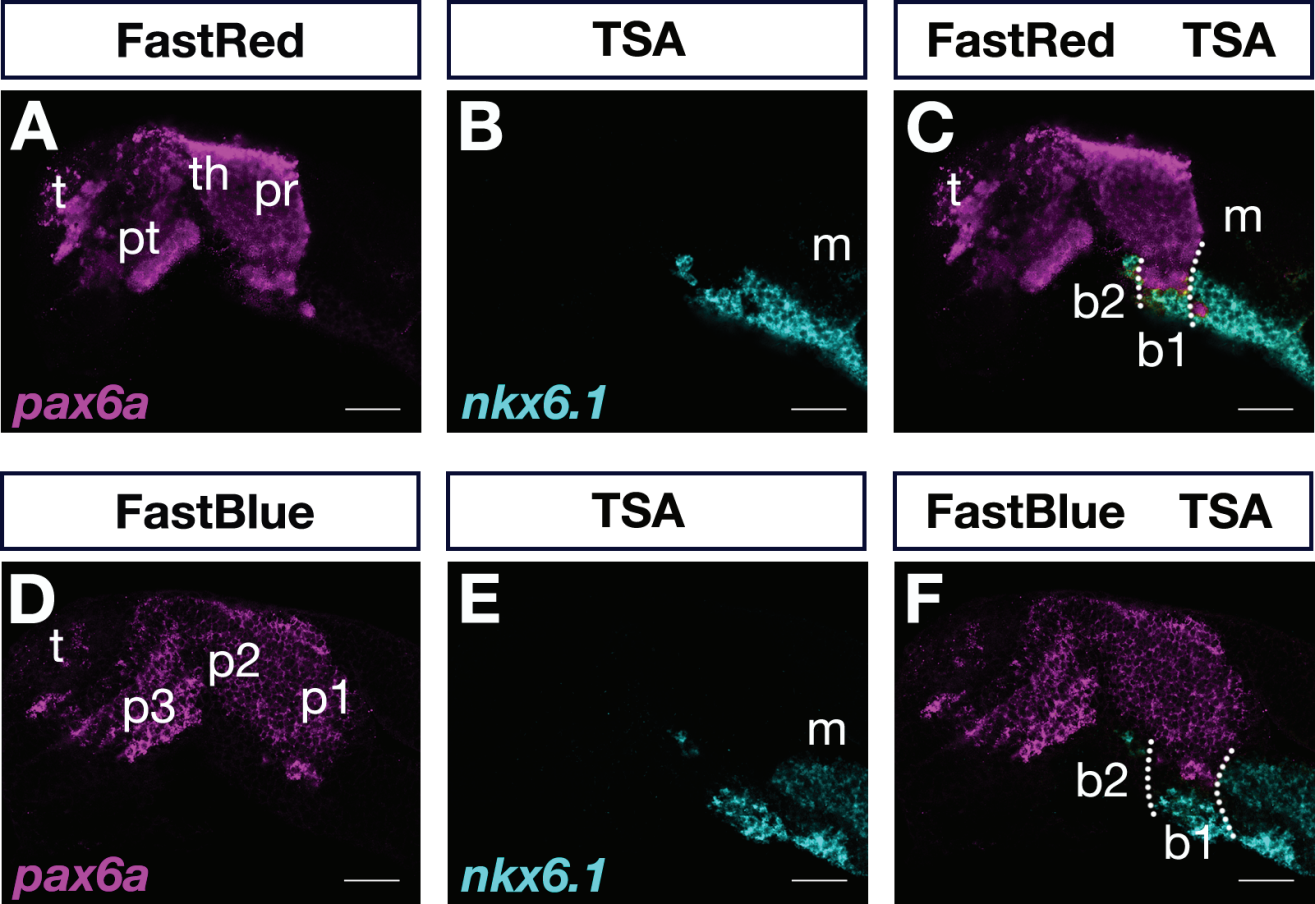
**Two-color FISH by combining POD and AP detection systems**. 24-hpf embryos were simultaneously hybridized to differently-labeled *pax6a *and *nkx6.1 *probes and visualized by sequential recording of TSA-FAM (B,C,E,F) and Fast Red (A,C) or Fast Blue (D,F). Single channels (A,B,D,E) and overlays of two channels (C,F) are shown. Detection channels for Fast Blue, Fast Red and TSA-FAM were >650 nm, >560 nm, and 505 nm to 545 nm, respectively. Dotted lines indicate boundaries of basal part of prosomere 1. Abbreviations: b1, basal part of prosomere 1; b2, basal part of prosomere 2; m, midbrain; p1, prosomere 1; p2, prosomere 2, p3 prosomere 3; pr, pretectum; pt, prethalamus; t, telencephalon; th, thalamus. Images were recorded on a LSM510 confocal microscope and false-colored in ImageJ. Scale bar is 50 μm.

We combined POD-TSA-FAM and AP-Fast Red (Figure 6A-C) or AP-Fast Blue (Figure 6D-F) detection systems to directly compare expression of *nkx6.1 *and *pax6a *in the same embryo. As described previously [10,11], expression of *pax6a *was confined to distinct domains in the telencephalon and diencephalon. In the alar diencephalon, expression of *pax6a *was confined to the prethalamus/prethalamic eminence, thalamus/epithalamus and pretectum (Figure 6A,D) corresponding to alar parts of prosomeres 3, 2, and 1 (p3, p2 and p1), respectively [31,32]. In addition, a separate *pax6a *cell group was found in the mantle layer of the pretectal basal plate. Expression of *nkx6.1 *extended in a longitudinal domain along the ventral brain (Figure 6B,E). From the midbrain tegmentum *nkx6.1 *expression extended rostrally into the diencephalic basal plate beneath the pretectal *pax6a *expression domain (Figure 6C,F). Interestingly, *nkx6.1 *expression precisely spared out the basal *pax6a *stripe confined to b1 (basal p1) and extended in a more ventral domain up to the b1/b2 boundary. In addition, few *nkx6.1*-positive cells were located more rostrally beneath the *pax6a*-positive thalamus and anterior to the basal *pax6a *domain (Figure 6C,F). These *nkx6.1 *cells were thus localized to b2 (basal p2). Our results indicate that *nkx6.1 *expression is consistent with prosomeric subdivisions of the basal zebrafish diencephalon [33]. Furthermore, basal forebrain expression of zebrafish *nkx6.1 *is very similar to that of orthologous genes. In amniotes, *Nkx6.1 *expression was reported to extend anteriorly into the region of basal p1 and p2 [34]. This suggests a conservation of *Nkx6.1 *gene expression in the basal diencephalon of anamniotes and amniotes. In conclusion, our results demonstrate the versatility of using Fast dye fluorescent detection in combination with POD-TSA-FAM in two-color FISH in order to compare differently localized gene expression domains at high resolution (step-by-step protocol, Additional file 1).

When comparing Fast Red and Fast Blue fluorescence signals we noted a sharp localization of the Fast Blue signal that allowed cellular resolution of transcript visualization (Figure 5A, 6D) while Fast Red sometimes produced a little less localized signal (Figure 4A, 6A). This suggested that Fast Red was more prone to diffusion of reaction products away from the site of enzymatic activity before precipitation, which may affect target resolution. In addition, prolonged Fast Red staining times could result in formation of spurious crystals (Figure 4A) and nonspecific background staining, while this was less of a problem with Fast Blue. In our hands, Fast Blue seemed slightly more sensitive than Fast Red as we obtained comparable signals within shorter staining times (55 versus 80 minutes for embryos in Figure 2D,H).

We showed that AP-based Fast Blue fluorescent detection in the far-red can be optimally combined with POD-based green fluorogenic TSA substrates excluding crosstalk and bleed-through in two-color experiments. Since Fast Blue emission is in the far-red, it can easily be combined with green and blue fluorochromes in two-channel fluorescence detection experiments. Fast Red, which has an emission spectrum closer to the green FAM resulting in potential bleed-through (Figure 4C), may be combined with cyan fluorochromes (e.g. Alexa Fluor 410). However, there is high UV and significant cyan autofluorescence in zebrafish embryos, which makes fluorescent signals in these channels more difficult to be resolved from background [20].

Current multiplex FISH methods [21-25] require serial rounds of enzymatic amplification and effective inactivation, potentially leading to progressive degradation of samples and reduced sensitivities of subsequent detection rounds. These difficulties may be circumvented by application of different enzymatic detection systems, as described here, allowing for one-step antibody detection. Another recent multiplexed FISH method used *in situ *hybridization chain reaction (HCR) for signal amplification [35]. HCR amplifiers operate independently in the same sample at the same time, allowing simultaneous detection of multiple target mRNAs, so that sample degradation of sequential detection procedures can be elegantly avoided. Another feature of this method is the use of short RNA probes and hairpins that are supposed to easily penetrate sample tissues. In this study, we optimized embryo permeabilization properties by hydrogen peroxide treatment and hybridization conditions by application of the viscosity-increasing polymer dextran sulfate for increased signal intensities. These measurements may be similarly beneficial for sample penetration and hybridization of short RNA probes and hairpins as described by [35] as well as locked nucleic acid (LNA)-modified DNA probes used for detection of miRNAs [36].

## Conclusions

One advantage of our protocol is that POD and AP coupled antibodies can be simultaneously applied, so that just one detection step is required and inactivation of antibody-reporter enzyme conjugates is omitted. Therefore, hands-on time and duration of the complete procedure are considerably reduced and the problem of false-positive signals by insufficient reporter enzyme inactivation is eliminated. When dextran sulfate was included in the hybridization mixture and hydrogen peroxide permeabilized embryos were used the staining time to achieve a strong signal was significantly shortened, which was advantageous for minimizing upcoming background and improving signal-to-noise ratio.

Since POD activity is rather quickly quenched by excess substrate, less abundant transcripts can often not be efficiently visualized despite tyramide signal amplification [19]. AP-Fast Blue fluorescent detection may provide a helpful alternative, as no enzyme inactivation is associated with the AP substrate reaction. Therefore, signal development can be extended at a high signal-to-noise ratio resulting in stronger signal intensities. The applicability of Fast Blue in fluorescent RNA visualization increases the number of possible substrate combinations to choose from. This may allow improved adjustment of detection systems and substrate combinations according to individual requirements of a specific two-color FISH experiment (Table 1). In addition, Fast Red and Fast Blue permit colorimetric as well as fluorescent transcript visualization, so that both types of data can be photographically documented and compared. This can be of advantage as fluorescence detection alone lacks histological context. Thus, our protocol provides a useful alternative method to study expression patterns in relation to each other. We applied our method for comparison of expression domains of neural specific regulatory genes, but this protocol will also be useful for two-color detection of transcripts in other tissues or organs and may be adapted for use in various model organism aside zebrafish.

**Table 1:** Application of different detection systems in two-color WISH

Detection System 1	Detection System 2	Applications
POD-TSA- DyLight633	POD-TSA-FAM	▪ Routinely used for fluorescent co-localization analysis of two mRNAs at cellular resolution ▪ If one of the two probes cannot be detected by POD-TSA, combine POD and AP detection

POD-TSA-FAM	AP-Fast Red	▪ If one probe cannot be visualized by POD-TSA this probe may be detected by AP-Fast Red allowing for long staining times with high signal-to-noise-ratios ▪ Combine fluorescence and DIC optics, if histological context is required ▪ Fast Red is preferably used for the more locally distributed mRNA

POD-TSA-FAM	AP-Fast Blue	▪ If there is significant bleed-through between TSA-FAM and AP-Fast Red, AP-Fast Blue as second detection system may be applied instead ▪ If the weaker probe cannot be detected by TSA-FAM or Fast Red, prolonged Fast Blue staining may be helpful for visualization ▪ Combine fluorescence and DIC optics, if histological context is required ▪ Fast Blue is preferably used for the more locally distributed mRNA

AP-Fast Blue	AP-Fast Red	▪ For fluorescent visualization of non-overlapping expression domains ▪ For colorimetric detection of two mRNAs ▪ Permits long term storage of stained embryos

AP-Fast Red	AP-BCIP/NBT	▪ Routinely used for colorimetric co-distribution analysis of two mRNAs ▪ Overlap of two mRNA distributions may be difficult to visualize at cellular resolution (for this purpose sectioning may be required) ▪ Combination of fluorescent and chromogenic detection is possible ▪ Permits long-term storage of stained embryos

## Methods

### Fish housing

Zebrafish were kept at 26°C in an aquaria facility from Schwarz Aquarienbau (Göttingen, Germany) under a 10 hour dark 14 hour light cycle. Embryos were obtained through natural mating in a 1 liter breeding trap, raised at 28.5°C and staged in hours-post-fertilization (hpf). Experiments were performed using only fixed specimen. All experiments were in accordance with ethical permits by Stockholms södra djurförsöksetiska nämnd (Stockholm south ethical council) and jordbruksverket (Swedish board of agriculture).

### RNA probes

Zebrafish *sim1a *[37], *shha *[38], *pax6a *[39] and *nkx6.1 *[40] cDNA plasmids were kindly provided by Fabrizio Serluca, Denis Duboule, Andreas Püschel and Judith Eisen, respectively. Digoxigenin and dinitrophenol-labeled [41] antisense mRNA probes were generated by T7/T3 *in vitro *transcription [5]. In order to avoid background staining unincorporated nucleotides were removed from the probe preparation. We routinely used column purification of RNA probes [8] according to the manufacturer's instructions (Omega Bio-Tek: Norcross, GA, USA R6249-02).

### Embryo pretreatment and hybridization

Embryos were fixed in 4% paraformaldehyde at 4°C overnight and stored in methanol at -20°C. 24-hpf embryos were permeabilized in 2% hydrogen peroxide in methanol for 20 minutes (min) at room temperature (RT) and stepwise rehydrated to PBST (phosphate buffered saline, 0.1% Tween-20 pH 7.3). After rehydration, embryos were further permeabilized by a 10-min proteinase K treatment and postfixed for 20 min with 4% paraformaldehyde at RT. Prehybridization, probe hybridization and washes were performed as described [5] except for addition of 5% dextran sulfate to the hybridization buffer. In two-color experiments, digoxigenin- and dinitrophenol-labeled probes were mixed together in hybridization buffer at the appropriate concentrations and hybridized simultaneously.

### Sequential alkaline phosphatase detection using Fast dyes

Hybridized embryos were blocked with 8% sheep serum (Sigma: St. Louis, MO, USA S2263) in PBST for at least 1 hour at RT. The digoxigenin probe was detected by incubation for 2 or more hours at RT (or at 4°C overnight) with anti-digoxigenin F_AB _fragments (Roche Scandinavia: Bromma, Sweden 11093274910) conjugated to AP diluted 1:4500. Transcripts were visualized in 1 mg/ml Fast Red TR and 0.4 mg/ml naphtol-AS-MX-phosphate (NAMP) in 0.1 M Tris-HCl pH 8.2. Before the second detection round, the antibody-AP conjugate was inactivated by 0.1 M glycine-HCl pH 2.2 treatment for 10 min at RT [8]. The dinitrophenol-labeled probe was detected by incubation for 2 or more hours at RT (or at 4°C overnight) with anti-dinitrophenol antibodies coupled to AP (Vector laboratories: Burlingame, CA, USA MB-3100) diluted 1:1000. The second mRNA probe was visualized in 0.25 mg/ml Fast Blue BB (Sigma F3378) and 0.25 mg/ml NAMP (Sigma N5000) in SB8.2 (0.1 M Tris-HCl pH 8.2, containing 50 mM MgCl_2_, 100 mM NaCl, 0.1% Tween-20) [4,26]. Orders of probe label, antibody detection and substrates can be exchanged according to individual requirements.

### Combination of AP and POD detection

Hybridized embryos were blocked with 8% sheep serum in PBST for at least 1 hour at RT. The digoxigenin and dinitrophenol labeled probes were simultaneously detected by incubation for 2 or more hours at RT (or at 4°C overnight) in a mixture of anti-digoxigenin POD fragments (Roche 11207733910) diluted 1:500 and anti-dinitrophenol AP conjugates 1:1000 (Vector laboratories MB-3100). Alternatively, embryos were incubated in a mixture of anti-digoxigenin AP F_AB _fragments (Roche 11093274910) diluted 1:4500 and anti-dinitrophenol POD (Perkin Elmer: Waltham, MA, USA TSA Plus DNP System NEL747A001KT) conjugates diluted 1:100. For visualization of the two different transcript probes, the POD-TSA reaction was performed first followed by AP-Fast Red or AP-Fast Blue staining. Stained embryos were washed by short rinses in TNT (100 mM Tris-HCl pH 7.5, 150 mM Sodium chloride, 0.1% Tween-20) and PBST.

### TSA reaction

Embryos were washed two times in 100 mM borate pH 8.5, 0.1% Tween-20 and incubated in freshly prepared TSA reaction buffer (100 mM borate pH 8.5, 2% dextran sulfate, 0.1% Tween-20, 0.003% H_2_O_2_) containing bench-made FAM tyramide reagent at a 1:250 dilution [25,42]. The TSA reaction was allowed to run for maximal 30 min protected from light and without agitation. Samples were rinsed thoroughly four times in PBST. For each washing step the tubes were inverted several times.

### AP substrates

BCIP 5-bromo-4-chloro-3-indolylphosphate (AppliChem: Darmstadt, Germany A1117), NBT nitro-bluetetrazolium chloride (AppliChem A1243), and Fast Blue BB 4-benzoylamino-2,5-diethoxybenzenediazonium chloride hemi[zinc chloride] salt (Sigma F3378) were dissolved in dimethyl formamide and kept as 50 or 100 mg/ml stock solutions at -20°C. NAMP 3-hydroxy-2-naphthoic acid 2,4-dimethylanilide phosphate (Sigma N5000) was kept as a 50 or 100 mg/ml stock in dimethyl sulfoxide at -20°C. Staining solutions were prepared just prior to use. Two separate solutions of 500 μg/ml Fast Blue and 500 μg/ml NAMP in SB8.2 were prepared first. The two double-concentrated solutions were then carefully mixed to achieve the final working concentration of 250 μg/ml per substrate. Fast Red/NAMP was obtained in tablet form (Sigma tablet set F4648) and prepared according to the manufacturer's instructions. The Fast Red working solution was filtered to remove non-dissolved substrate particles.

### Microscopic visualization

Embryos were mounted in 75% glycerol and viewed under a Axioplan2 microscope (Carl Zeiss: Jena, Germany). Photographs were taken with an Axiocam color digital camera (Figure 1, 2 and 3). For fluorescence detection of Fast Red and Fast Blue, rhodamine and far-red filters were used, respectively. For high-resolution confocal microscopy embryos were mounted in 75% glycerol in TNT pH 8 containing 1% low-melting agarose (Figure 4, 5 and 6). Fluorescent signals were recorded on a Carl Zeiss LSM510 confocal microscope using the 633 nm laser lines for Fast Blue, 543 nm for Fast Red, and 488 nm for TSA-FAM for excitation. Fast Blue, Fast Red and TSA-FAM fluorescence emissions were detected using high pass filters LP505, LP560 and LP650 that pass wavelengths greater than 505 nm, 560 nm and 650 nm to the detector, respectively. Images were processed in opensource ImageJ software to adjust outliers and intensity levels before running a Gaussian smoothening filter. Black-and white pictures were false-colored with the help of ImageJ software. Figure panels were assembled using Adobe Photoshop software.

## Abbreviations

AP: alkaline phosphatase; FAM: carboxyfluorescein; FISH: fluorescent *in situ *hybridization; hpf: hours-post-fertilization; NAMP: naphtol-AS-MX-phosphate; POD: horseradish peroxidase; TSA: tyramide signal amplification; WISH: whole-mount *in situ *hybridization

## Competing interests

The authors declare that they have no competing interests.

## Authors' contributions

GL and IS did the experiments. GH designed the project, GH wrote the manuscript and GL designed the figures. All three authors contributed to finalizing the manuscript, read and approved the final version of the manuscript.

## Supplementary Material

Additional file 1 **Two-color FISH protocol combining POD and AP detection systems**. This file contains a step-by-step protocol for use in the laboratory. Click here for file
