# Supplementary material for: Two-color fluorescent in situ hybridization in the embryonic zebrafish brain using differential detection systems
Source: BMC Dev Biol. 2011 Jul 4;11:43. doi: 10.1186/1471-213X-11-43 (PMC3141750; doi:10.1186/1471-213X-11-43)
Supplement: Additional file 1 — Two-color FISH protocol combining POD and AP detection systems. This file contains a step-by-step protocol for use in the laboratory. [file 1471-213X-11-43-S1.PDF]

Additional file 1: Two-color FISH protocol combining POD and AP detection systems

1.1 Embryo Pre-treatment

1.) Transfer dechorionated embryos of the desired developmental stage to a 2 mL microcentrifuge tube. Fix embryos in 1 mL of 4% paraformaldehyde (PFA) for 24 h at 4°C.

2.) Wash embryos four times for 5 min with 2 mL phosphate buffered saline containing 0.1% Tween-20 (PBST) and subsequently transfer embryos to 100% methanol (MeOH). Exchange the 100% MeOH after 5 min and incubate embryos at -20°C for at least 30 min. Embryos may be kept in MeOH at -20°C for long-term storage.

3.) Permeabilize embryos in 2% hydrogene peroxide (H_2O_2) in 100% MeOH for 20 min. Successively rehydrate embryos through 5 min washing steps in 75% MeOH / 25% PBST, 50% MeOH / 50% PBST, 25% MeOH/ 75% PBST followed by two PBST washes.

4.) Embryos with an enclosed yolk (tailbud stage and older) have to be treated with proteinase K to further enhance accessibility of probes. The optimal digestion time for a distinct embryo sample has to be determined experimentally. Digest embryos in proteinase K (10 µg/mL PBST) at RT and stop the reaction by rinsing twice with glycine (2 mg/mL PBST). Postfix embryos in 4% PFA for 20 min at RT and wash four times for 5 min in PBST. Transfer embryos to 1 mL pre-hybridization buffer (Hb4). Embryos can be stored in Hb4 at -20°C or processed immediately for hybridization.

5.) Transfer up to 25 embryos to 200 µl of Hb4 in a 2 mL microcentrifuge tube. Incubate in a water-bath at 60°C for 1 h for pre-hybridization.

1.2 Hybridization

1.) Prepare the probe-mix by adding dinitrophenol and digoxigenin labeled RNA probes in 150 µl Hb4 containing 5% dextran sulfate (Hb4D5). Denature the probe-mix for 5 min at 80°C and equilibrate to 60°C. Carefully remove pre-hybridization solution from embryo sample and add the pre-warmed probe-mix. Incubate embryos at 60°C in a water-bath overnight (min. 15h).
2.) For all post-hybridization washes the solutions have to be prewarmed to 60°C.
3.) Wash embryos twice with 0.2 mL of 50% formamide (FA) in 2xSSCT (saline sodium citrate) for 30 min.
4.) Wash once with 2 mL 2xSSCT for 15 min.
5.) Wash twice with 2 mL 0.2xSSCT for 30 min.
6.) Add PBST and let cool down to RT.

1.3 Antibody Detection

The two differently-labeled RNA probes are simultaneously detected by a mixture of sheep-anti-digoxigenin-POD Fab fragments (Roche 11207733910) and rabbit-anti-dinitrophenyl-AP antibody (Vector laboratories MB-3100) diluted in blocking solution 1:500 and 1:1000, respectively.

1.) Incubate embryos in 100 µL of 8% blocking solution for 1 h at RT on a gently rocking table.
2.) Thoroughly remove the blocking solution and add the antibody mixture. Incubate overnight at 4°C without agitation.
3.) To remove unbound antibody wash six times with PBST for 20 min at RT under gentle agitation.

1.4 Fluorogenic detection

FAM staining:

In order to avoid a decline in signal-to-noise ratio the reaction time should not exceed 30 min. The use of an accelerator for the peroxidase reaction significantly enhances the TSA reaction. Use 4-iodophenol (Fluka 58020) or vanillin (Sigma V110-4) at a concentration of 0.15 mg/ml and 0.45 mg/ml, respectively (*see* Lauter et al., 2011 Neural Dev. 6:10). Higher concentrations maybe used to further increase signal intensity, although adverse effects on the signal-to-noise ratio should be kept in mind. Be **CAREFUL** when working with 4-iodophenol, as it is a highly aggressive substance and should **ONLY** be handled with proper safety measures (gloves, lab coat, eyeshields, faceshields etc.) under a fume hood at all time (even when highly diluted).

1.) Freshly prepare TSA reaction buffer in a 2 mL microcentrifuge tube. Prepare at least 1 mL (calculate 100 µL per reaction).
2.) For 1 mL TSA reaction buffer combine 100 µl of 1 M borate-buffer pH 8.5, 40 µL of 50% dextran sulfate, 10 µL of 10% Tween-20, 6 µL of 0.5% H_2O_2 and add an POD accelerator. Adjust the volume with distilled H_2O to 1 mL.
3.) Addition of 4-iodophenol leads to a cloudy solution. Mix the TSA reaction buffer well by pipetting up and down until it has an opaque appearance.
4.) Dilute bench-made FAM-tyramide 1:250 (*see* Lauter et al., 2011 Neural Dev. 6:10) with the TSA reaction buffer and mix well by pipetting.
5.) Rinse embryos twice in 100 mM borate pH 8.5 plus 0.1% Tween-20. Thoroughly remove borate solution and add 90 µL of the FAM-tyramide solution to the embryos. Gently mix by pipetting using a cut tip. Incubate for 15 – 30 min at RT protected from light and without agitation.
6.) Rinse sample four times with PBST. For each washing step fill the entire tube with PBST and close it, invert it several times, wait until the embryos sink down to the bottom and then remove the excess buffer carefully.

1.5 Chromogenic Detection

Staining solutions are prepared freshly just prior to use. Chromogenic detection of the second RNA probe can immediately follow the FAM-TSA detection procedure. We routinely conduct the TSA-POD reaction first, since the subsequent AP-Fast dye staining can be performed for prolonged time with a high signal-to-noise ratio to achieve optimal signal strength for the second probe.

Fast Blue staining:

1.) Wash twice with PBST for 5 min at RT.
2.) Incubate in staining buffer (SB8.2) for 5 min at RT.
3.) Prepare a 0.5 mg/mL Fast Blue BB solution in SB8.2. Prepare a separate 0.5 mg/mL naphtol-AS-MX (NAMP) solution in SB8.2.
4.) To avoid localized precipitation of substrate solution, add the NAMP solution drop-wise into the Fast Blue BB solution under constant mixing on a vortex. This will result in a final working solution of 0.25 mg/mL of each substrate component.
5.) Cover embryos with staining solution and incubate in the dark.
6.) Regularly check the staining.
7.) Exchange staining solution with fresh one if substrate starts precipitating.
8.) Stop staining by washing four times for 5 min in TNT and twice in PBST.

Fast Red staining:

1.) Wash twice with PBST for 5 min at RT.
2.) Wash with TNT for 5 min at RT.
3.) Dissolve a buffer tablet (0.1 M Tris-HCl pH 8.2) from the Fast Red TR/NAMP Alkaline Phosphatase Substrate Tablets Set (Sigma F4648) in 1 mL distilled H_2O by vortexing. Drop one tablet of Fast Red TR/NAMP into the Tris buffer and dissolve it by vortexing.
4.) Pass the staining solution through a 0.2 μm filter to remove non-dissolved substrate particles.
5.) Cover embryos with staining solution and incubate in the dark.
6.) Regularly check the staining.
7.) Stop staining by washing four times for 5 min in TNT and twice in PBST.

1.6 Buffer recipes

Blocking solution: 8% normal sheep serum in 1x PBST

HB4: 50% deionized formamide, 5xSSC, 5 mg/mL torula RNA (Sigma R6625), 50 µg/mL heparin sodium salt, 0.1% Tween-20

HB4D5: Hb4, 5% (v/v) dextran sulfate

1xPBS: 8% (w/v) NaCl, 0.2% (w/v) KCl, 16 mM Na_2HPO_4, 4 mM NaH_2PO_4, pH 7.3

PBST: 1xPBS, 0.1% (v/v) Tween-20

PFA: 4% paraformaldehyde in 1xPBS, pH 7.3

SB8.2: 100 mM Tris pH 8.2, 100 mM NaCl, 50 mM $MgCl_2$, 0.1% Tween-20

Prepare freshly since $MgCl_2$ will precipitate as $Mg(OH)_2$ at alkaline pH.

20xSSC: 3M NaCl, 300 mM trisodium citrate, pH 7.0

2xSSCT: 2xSSC, 0.1% Tween-20

0.2xSSCT: 0.2xSSC, 0.1% Tween-20

TNT: 100 mM Tris pH 7.5, 150 mM NaCl, 0.1% Tween-20

TSA reaction buffer: 100 mM borate buffer pH 8.5, 2% dextran sulfate, 0.1% Tween-20, 0.003% H_2O_2
